# An MRI-based radiomics nomogram for differentiating spinal metastases from multiple myeloma

**DOI:** 10.1186/s40644-023-00585-4

**Published:** 2023-07-24

**Authors:** Shuai Zhang, Menghan Liu, Sha Li, Jingjing Cui, Guang Zhang, Ximing Wang

**Affiliations:** 1 Shandong Provincial Hospital Affliated to Shandong First Medical University, Shandong, China; 2 Depertment of Health Management, The First Affiliated Hospital of Shandong First Medical University, Shandong, China; 3 Cheeloo College of Medicine, Shandong University, Shandong, China; 4 United Imaging Intelligence Co., Ltd, Beijing, China; 5 Department of Radiology, Shandong Provincial Hospital, Shandong First Medical University, No.324 Jingwu Road, Jinan, Shandong 250021 China; 6 Depertment of Health Management, The First Affiliated Hospital of Shandong First Medical University, No. 16766, Jingshi Road, Jinan, Shandong 250014 China

**Keywords:** Radiomics, Multiple myeloma, Magnetic resonance imaging, Spine

## Abstract

**Background:**

Spinal metastasis and multiple myeloma share many overlapping conventional radiographic imaging characteristics, thus, their differentiation may be challenging. The purpose of this study was to develop and validate an MRI-based radiomics nomogram for the differentiation of spinal metastasis and multiple myeloma.

**Materials and methods:**

A total of 312 patients (training set: n = 146, validation set: n = 65, our center; external test set: n = 101, two other centers) with spinal metastasis (n = 196) and multiple myeloma (n = 116) were retrospectively enrolled. Demographics and MRI findings were assessed to build a clinical factor model. Radiomics features were extracted from MRI images. A radiomics model was constructed by the least absolute shrinkage and selection operator method. A radiomics nomogram combining the radiomics signature and independent clinical factors was constructed. And, one experienced radiologist reviewed the MRI images for all case. The diagnostic performance of the different models was evaluated by receiver operating characteristic curves.

**Results:**

A clinical factors model was built based on heterogeneous appearance and shape. Twenty-one features were used to build the radiomics signature. The area under the curve (AUC) values of the radiomics nomogram (0.853 and 0.762, respectively) were significantly higher than that of the clinical factor model (0.692 and 0.540, respectively) in both validation (*p* = 0.048) and external test (*p* < 0.001) sets. The AUC values of the radiomics nomogram model were higher than that of radiologist in training, validation and external test sets (all *p* < 0.05). Moreover, no significant difference in AUC values of radiomics nomogram model was found between the validation set and external test set (*p* = 0.212).

**Conclusion:**

The radiomics nomogram can differentiate spinal metastasis and multiple myeloma with a moderate to good performance, and may be as a valuable method to assist in the clinical diagnosis and preoperative decision-making.

**Supplementary Information:**

The online version contains supplementary material available at 10.1186/s40644-023-00585-4.

## Background

Metastasis and multiple myeloma are two common malignant diseases involving the spine [1, 2]. These conditions often result in pathological fractures with associated neurological complications, especially in the elderly [3]. In addition, the spinal metastasis shares many overlapping conventional radiographic imaging characteristics with multiple myeloma, especially presenting with multiple osteolytic features [4]. However, their treatment decision and prognosis are significantly different. Thus, accurate distinction between spinal metastasis and multiple myeloma is vital for assessment of prognosis and choice of suitable treatment.

Previous reports attempted to distinguish spinal metastasis and multiple myeloma with the use of MRI conventional and functional imaging [5, 6]. By using diffusion weight imaging, Xing et al. [5] tried to differentiate spinal metastasis from multiple myeloma. Nevertheless, these studies were likely affected by an individual observer, thus, their diagnostic efficacy needs to be improved.

Nowadays, the increasing attention is being paid to radiomics studies, which is a novel non-invasive technique. Radiomics can extract more features from conventional images and identify subtle changes beyond those detectable by visual assessment [7]. Previous studies have shown that radiomics had good value for the tumor diagnosis, grading, and prognostic evaluation [8–10]. Chianca et al. [11] reported the usefulness of radiomics analysis for distinguishing benign from malignant tumors in the spine. A previous study showed that the radiomics model could well differentiate metastasis from multiple myeloma based on MRI [12]. However, it mainly focused on influence of features number and the limited sample were collected from one center. So far comparative studies based on radiomics analysis in musculoskeletal radiology are limited and more radiomics studies are necessary to provide a more comprehensive diagnosis analysis.

The purpose of this study was to develop and validate a radiomics nomogram incorporating radiomics signatures and clinical factors for differentiation of metastasis and multiple myeloma.

## Materials and methods

### Patients

Institutional review board approval was obtained, and patient informed consent was waived due to the retrospective nature of this study.

A total of 312 patients diagnosed with spinal metastasis (n = 196) and multiple myeloma (n = 116) at three hospitals between January 2016 to July 2021 were included. The inclusion criteria were as follows: (1) patients diagnosed with multiple myeloma according to the International Myeloma Working Group Diagnostic Criteria [13] or metastatic tumors in the spine confirmed by pathological information; (2) presence of spinal lesions on MRI, including T1-weighted imaging (T1WI) and fat suppressed T2-weighted imaging (FS-T2WI) sequences. The exclusion criteria were as follows: (1) history of tumor therapy before MRI examination; (2) poor MRI image quality due to scanning conditions and lesion characteristics, such as vertebral fractures affecting the image signal; (3) presence of non-primary tumors outside the bone.

**Fig. 1:**
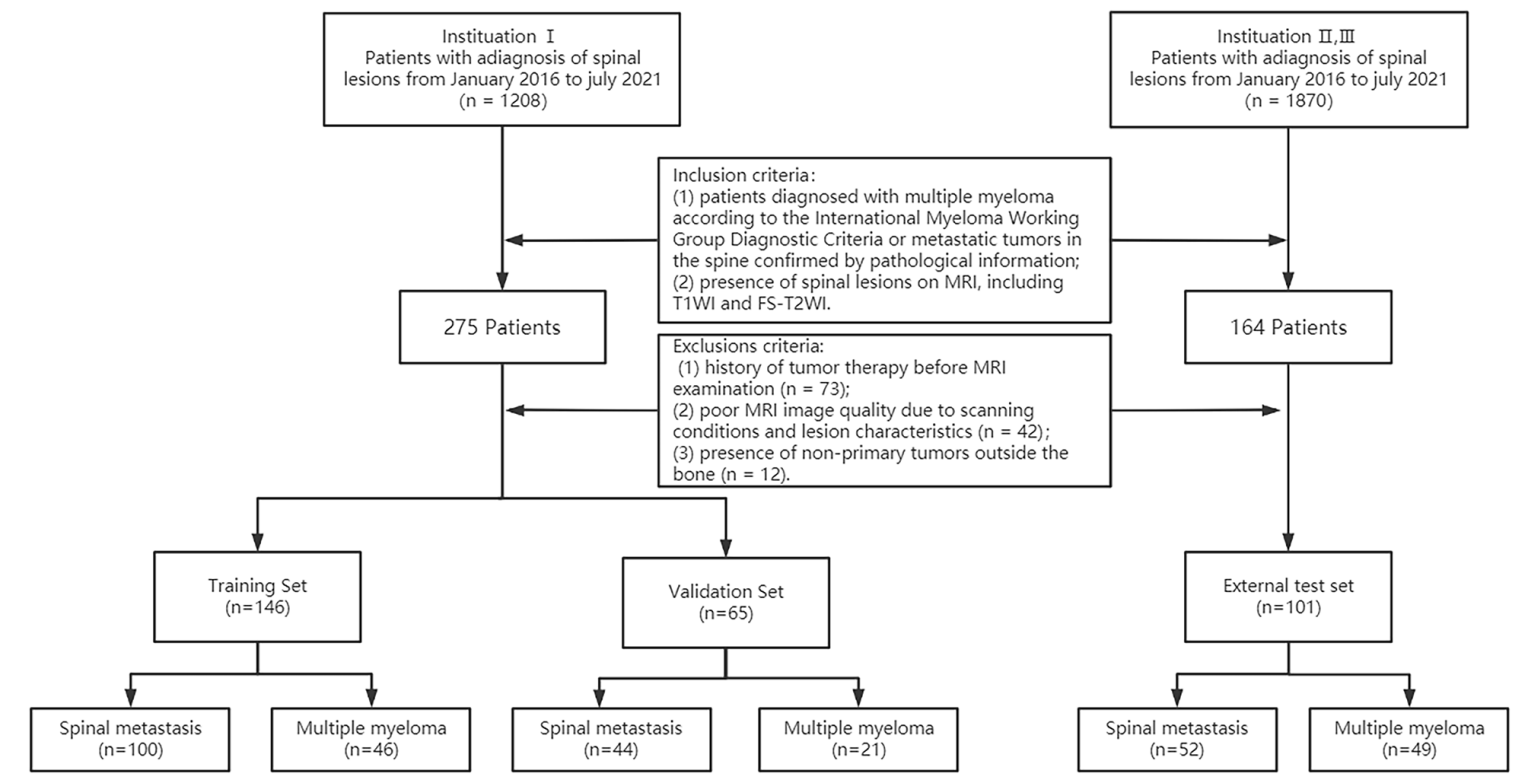
Flow diagram of the study

Finally, 211 patients (mean age ± standard deviation, 60.1 ± 10.7 years; 127 men), i.e., 144 with spinal metastasis and 67 with multiple myeloma from Shandong Provincial Hospital Affiliated to Shandong First Medical University were randomly assigned to the training and validation sets at a ratio of 7:3. An external set contained 101 patients (mean age, 63.2 ± 10.9 years; 64 men), including 40 patients (mean age, 65.1 ± 10.8 years; 23 men), i.e., 20 with spinal metastasis and 20 with multiple myeloma from Shandong Province Yuhuangding Hospital, and 61 patients (mean age, 62.0 ± 10.9 years; 41 men), i.e., 32 with spinal metastasis and 29 with multiple myeloma from Shandong Province Qianfoshan Hospital. The flowchart for selecting the study population is shown in Fig. 1.

### MRI protocol

The MRI was performed on 3.0 Tesla MR scanners (Magnetom Skyra, Siemens Healthineers; Prisma, Siemens Healthineers; Magnetom Spectra, Siemens Healthineers) and followed a similar protocol. The scanning sequence parameters for T1WI were as follows: repetition time (TR), 400–800 ms; echo time (TE), 10–30 ms; field of view, 320 × 320 mm; slice thickness, 3.0 mm; slice spacing, 1.0 mm. Settings for FS-T2WI were as follows: TR, 2000-4200ms; TE, 70–110 ms; field of view, 320 × 320 mm; slice thickness, 3.0 mm; slice spacing, 1.0 mm.

### Development of clinical factor model

Clinical factors including age, gender, diameter, margin (well-defined/ill-defined), heterogeneous appearance (present/absent), shape (regular/irregular), and number of lesions (single/multiple) were obtained. The heterogeneous appearance was defined as necrosis, cystic areas, hemorrhage and calcification in the lesion. In order to standardize the definition of lesion shape, all round or oval tumors were considered as regularly shaped; all others were considered as irregularly shaped. The independent samples t tests and chi-square or Fisher’s exact tests were applied to compare the differences of the clinical factors between the spinal metastasis and multiple myeloma. Next, a multiple logistic regression analysis was performed to build the clinical factor model by the above results with *p* < 0.05. Odds ratios as estimates of relative risk with 95% confidence intervals were calculated for each independent factor.

### Image segmentation and radiomics feature extraction

Tumor segmentation and feature extraction were performed with a post-processing platform (Lianying Medical Technology Co., Ltd). Segmentation was performed on the MR images including T1WI and FS-T2WI. Regions of interests (ROIs) were manually selected and delineated in the largest lesion. If there was extra-osseous extension of the lesion, it was also contoured. The necrosis, cystic areas, hemorrhage and calcification in the lesion were included. Contouring was drawn within the border of the tumor and the adjacent normal tissues were not covered. Two radiologists (SZ and MHL, with 7 and 8 years of experience in tumor radiology, respectively) independently performed the ROI segmentation and were blinded to the clinical data.

### Development of radiomics signature and radiomics nomogram, evaluation of radiologist

The radiomics features were selected by intraclass correlation coefficients (ICCs) > 0.75, significance in one-way analysis of variance (ANOVA), and finally using select_k_best method and the least absolute shrinkage and selection operator (LASSO) regression model in the training cohort. Then, the final selected features were applied to build a radiomics signature model. A radiomics score (known as radiomics signature) was calculated by a linear combination of selected radiomics features whose weights were based on logistic regression. Finally, a radiomics nomogram was developed by combining the significant variables of the clinical factors and the radiomics signature.

One radiologists (XMX, 14 years of experience in musculoskeletal radiology) independently reviewed MRI examinations for all cases, for purposes of comparison with the radiomics model.

### Assessment of the performance of different models

The diagnostic performance of four models (the clinical factor model, the radiomics signature model, the radiomics nomogram model, and one experienced radiologist) for identification of spinal metastasis and multiple myeloma was evaluated from the area under the curve (AUC) of the receiver operator characteristic (ROC) curve in the training, validation and external test sets at the same time. In order to assess the clinical usefulness of nomogram, a decision curve analysis (DCA) was performed by calculating the net benefits.

### Statistical analysis

Univariable analysis was used to compare differences in the clinical factors between the spinal metastases and multiple myeloma, with an independent samples t-test for quantitative data and chisquare or Fisher’s exact tests for qualitative data, as appropriate. One-way ANOVA was used to compare the value of each radiomics feature for the differentiation of spinal metastasis and multiple myeloma. Differences in the AUC values of different models were estimated using the Delong test. The calibration of the nomogram was estimated by Hosmer-Lemeshow. The clinical usefulness of nomogram was assessed by DCA. Comparison of sensitivity, specificity, and accuracy of models were performed with the McNemar’s test. *p* < 0.05 indicated statistical significance. Statistical analysis was performed using SPSS (version 22.0, IBM) and R statistical software (version 3.3.3, https://www.r-project.org).

## Results

### Patient characteristics

Seventy-three patients with a history of tumor therapy before the MRI examination, 42 patients who had poor MRI image quality, and 12 patients with non-primary tumors outside the bone were excluded. Finally, a total of 312 patients (mean age, 61.1 ± 10.9 years; 191 men) with 196 spinal metastasis and 116 multiple myeloma were included in this study. The primary cancers of spinal metastasis included lung cancers (n = 94), breast cancer (n = 18), prostate cancer (n = 13), liver cancer (n = 13), renal cancer (n = 9), esophageal cancer (n = 9), stomach cancer (n = 8), rectal cancer (n = 8), colon cancer (n = 7), thyroid cancer (n = 5), pancreatic cancer (n = 4), thymic cancer (n = 3), nasopharyngeal carcinoma (n = 2), endometrial carcinoma (n = 2), and cervical cancer (n = 1).

### Clinical factors of the patients and construction of the clinical factor model

Tumor diameter, heterogeneous appearance and shape showed significant differences between the spinal metastasis and multiple myeloma (*p* < 0.05) in training set. The multiple logistic regression analysis showed that heterogeneous appearance (*p* = 0.010) and shape (*p* = 0.005) remained independent predictors in the clinical factor model. The clinical and radiological characteristics of tumors are described in Table 1.

**Table 1:** Clinical factors of the training, validation and external sets

Characteristics	Training set (n = 146)			Validation set (n = 65)			External test set (n = 101)		
	Metastasis(n = 100)	Myeloma (n = 46)	P	Metastasis (n = 44)	Myeloma (n = 21)	P	Metastasis (n = 52)	Myeloma (n = 49)	P
Age (year)	60.0 ± 11.2	59.8 ± 10.1	0.910	60.3 ± 10.4	61.1 ± 11.4	0.766	62.1 ± 12.8	64.4 ± 8.4	0.287
Gender (male/female)	60/40	26/20	0.692	27/17	14/7	0.679	32/20	32/17	0.694
Diameter (cm)	2.1 ± 1.0	1.7 ± 1.5	0.044	2.4 ± 1.6	1.6 ± 0.9	0.032	2.2 ± 0.9	1.5 ± 0.7	< 0.001
Margin (well-defined/ill-defined)	63/37	22/24	0.840	29/15	11/10	0.294	28/24	33/16	0.166
Heterogeneous appearance (present/absent)	76/24	22/24	0.001	36/8	10/11	0.005	39/13	38/11	0.763
Shape (regular/irregular)	17/83	22/24	< 0.001	6/38	12/9	0.001	15/34	7/45	0.037
Number of lesions (single/multiple)	8/92	5/41	0.572	4/40	3/18	0.527	5/47	4/45	0.798

Continuous variables are presented as mean± standard deviation, categorical data as numbers (n)

### Feature extraction, selection, and radiomics signature establishment

In total, 2818 radiomics features extracted from T1WI and FS-T2WI, 1534 features with ICCs from 0.75 to 1 were tested by ANOVA, revealing 1161 features with significant differences between spinal metastasis and multiple myeloma (*p* < 0.05). The select_k_best method was used to eliminate the redundant and irrelated features. The remaining 695 features were then included in the LASSO to select the most valuable features. Twenty-one features building the radiomics signature were finally selected by LASSO. The detailed 21 features are shown in Supplementary Table 1. A radiomics score was calculated using the formula: radiomics-score = Coefficient × Radiomics features. The coefficient and radiomics features are detailed in Supplementary Table 1.

### The radiomics nomogram establishment and assessment of the performance of different models

**Fig. 2:**
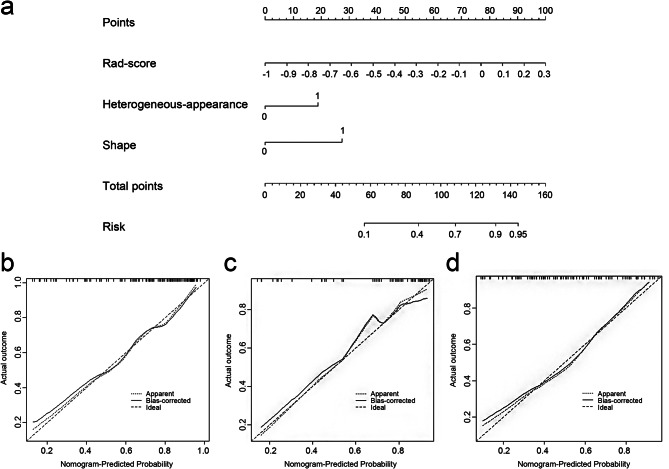
The radiomics nomogram and calibration curves for the radiomics nomogram. **(a)** The radiomics nomogram, combining rad-score, heterogeneous-appearance and shape, developed in the training set. Calibration curves for the radiomics nomogram in the training **(b)**, validation **(c)** and external test sets **(d)**. Calibration curve indicates the goodness-fit for the nomogram. The 45° dotted line represents an ideal prediction, and the other dotted line represents the predictive performance of the nomogram. A closer distance between two lines indicates better prediction. The solid line represents the bias corrected

The radiomics-score, heterogeneous appearance and shape were incorporated into the radiomics nomogram model (Fig. 2). The Fig. 2 showed radiomics nomogram had a good calibration by calibration curve and the Hosmer-Lemeshow test.

**Fig. 3:**
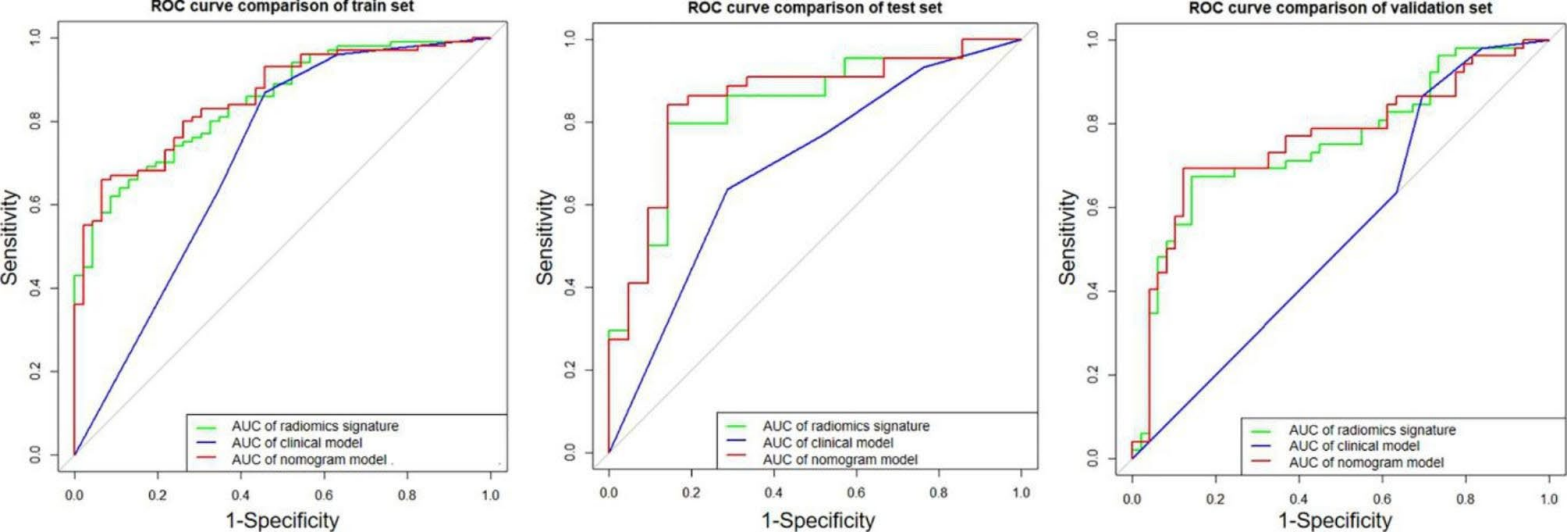
The receiver operating characteristic curves of the clinical factor model, the radiomics signature model, and radiomics nomogram model in the training **(a)**, validation **(b)** and external test **(c)** sets, respectively. In training set, the AUC values of radiomics nomogram was higher than radiomics signature (0.856 vs. 0.847, *p* = 0.342) and clinical factor model (0.856 vs. 0.715, *p* = 0.046). The AUC values of the radiomics nomogram (0.853 and 0.762, respectively) was significantly higher than that of the clinical factor model (0.692 and 0.540, respectively) in both the validation (*p* = 0.048) and external test (*p* < 0.001) sets

The diagnostic performances of the clinical factor model, radiomics signature, and radiomics nomogram are summarized in Table 2. The ROC curves of the three models for the training, validation and external test sets are shown in Fig. 3.

**Table 2:** Diagnostic performance of the clinical factor model, radiomics signature, radiomics nomogram, and one experienced radiologist for detection of spinal metastasis

Set	Model	AUC(95%CI)	Sensitivity(%)	Specificity(%)	Accuracy(%)
Training set	Clinical model	0.715(0.641–0.791)	87.0	54.3	76.7
	Radiomics signature	0.847(0.795–0.898)	75.0	73.9	74.7
	Radiomics nomogram	0.856(0.804–0.907)	73.0	76.1	74.0
	Radiologist	0.711 (0.630–0.783)	77.0	65.2	73.3
Validation set	Clinical model	0.692(0.531–0.730)	77.3	47.6	67.7
	Radiomics signature	0.743(0.668–0.861)	81.8	71.4	78.5
	Radiomics nomogram	0.853(0.764–0.919)	84.1	85.7	84.6
	Radiologist	0.613 (0.484–0.731)	75.0	47.6	66.2
External test set	Clinical model	0.540(0.517–0.651)	86.5	30.6	59.4
	Radiomics signature	0.754(0.595–0.748)	71.2	63.3	67.3
	Radiomics nomogram	0.762(0.605–0.751)	78.8	67.1	68.3
	Radiologist	0.632 (0.531–0.726)	67.3	59.2	63.4

AUC = area under the curve; CI = confidence interval

In the training set, the AUC values of radiomics nomogram were higher than radiomics signature (0.856 vs. 0.847, *p* = 0.342) and clinical factor model (0.856 vs. 0.715, *p* = 0.046). The AUC values of the radiomics nomogram (0.853 and 0.762, respectively) were significantly higher than that of the clinical factor model (0.692 and 0.540, respectively) in both the validation (*p* = 0.048) and external test (*p* < 0.001) sets. No significant difference (*p* = 0.265) in AUC values of radiomics signature model was found between the validation set (AUC, 0.743; 95%CI, 0.668–0.861) and external test set (AUC, 0.754; 95%CI, 0.595–0.748). And, no significant difference (*p* = 0.212) in AUC values of radiomics nomogram model was found between the validation set (AUC, 0.853, 95%CI, 0.764–0.919) and external test set (AUC, 0.762, 95%CI, 0.605–0.751). In external test set, the sensitivity (78.8% vs. 71.2%, *p* < 0.05), specificity (67.1% vs. 63.3%, *p* < 0.05), and accuracy (68.3% vs. 67.3%, *p* > 0.05) of nomogram model were higher than radiomics signature model.

**Fig. 4:**
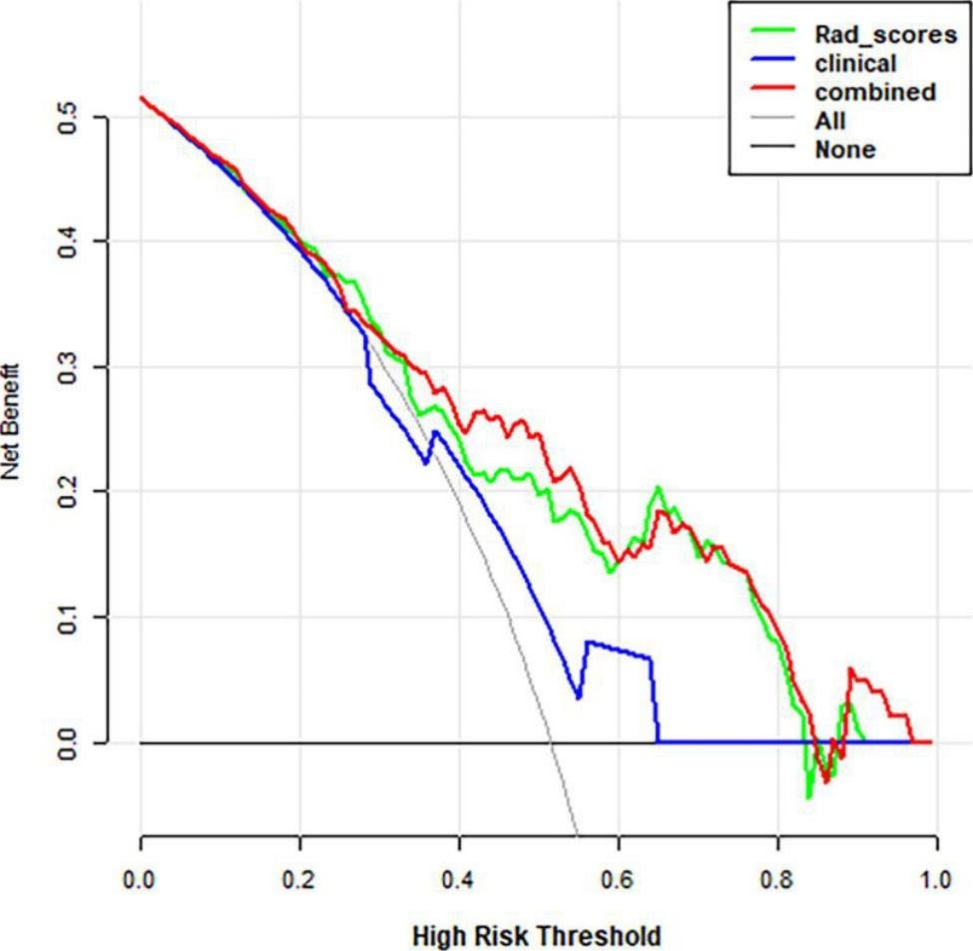
Decision curve analysis for three models. The y-axis indicates the net benefit; x-axis indicates threshold probability. The blue line, green line, and red line represent net benefit of the clinical factor, the radiomics signature, and the radiomics nomogram, respectively. The nomogram model had a higher net benefit in differentiating spinal metastasis and multiple myeloma than the other two models and simple diagnoses such as all spinal metastasis patients (gray line) or all multiple myeloma patients (black line), across the full range of threshold probabilities at which a patient would be diagnosed as spinal metastasis

The DCA for the three models is shown in Fig. 4. The decision curve analysis showed the radiomics nomogram had a higher overall net benefit in differentiating spinal metastasis and multiple myeloma than the clinical factor model across the most reasonable threshold probabilities.

### Comparison between the Radiomics Model and Radiologist

The AUC values were 0.711 (95%CI, 0.630–0.783), 0.613 (95%CI, 0.484–0.731), and 0.632 (95%CI, 0.531–0.726) respectively in training, validation, and external test sets for radiologist. The sensitivity, specificity, and accuracy, respectively, were 77.0%, 65.2%, and 73.3% in training set; 75.0%, 47.6%, and 66.2% in validation set, and 67.3%, 59.2%, and 63.4% in external test set.

**Fig. 5:**
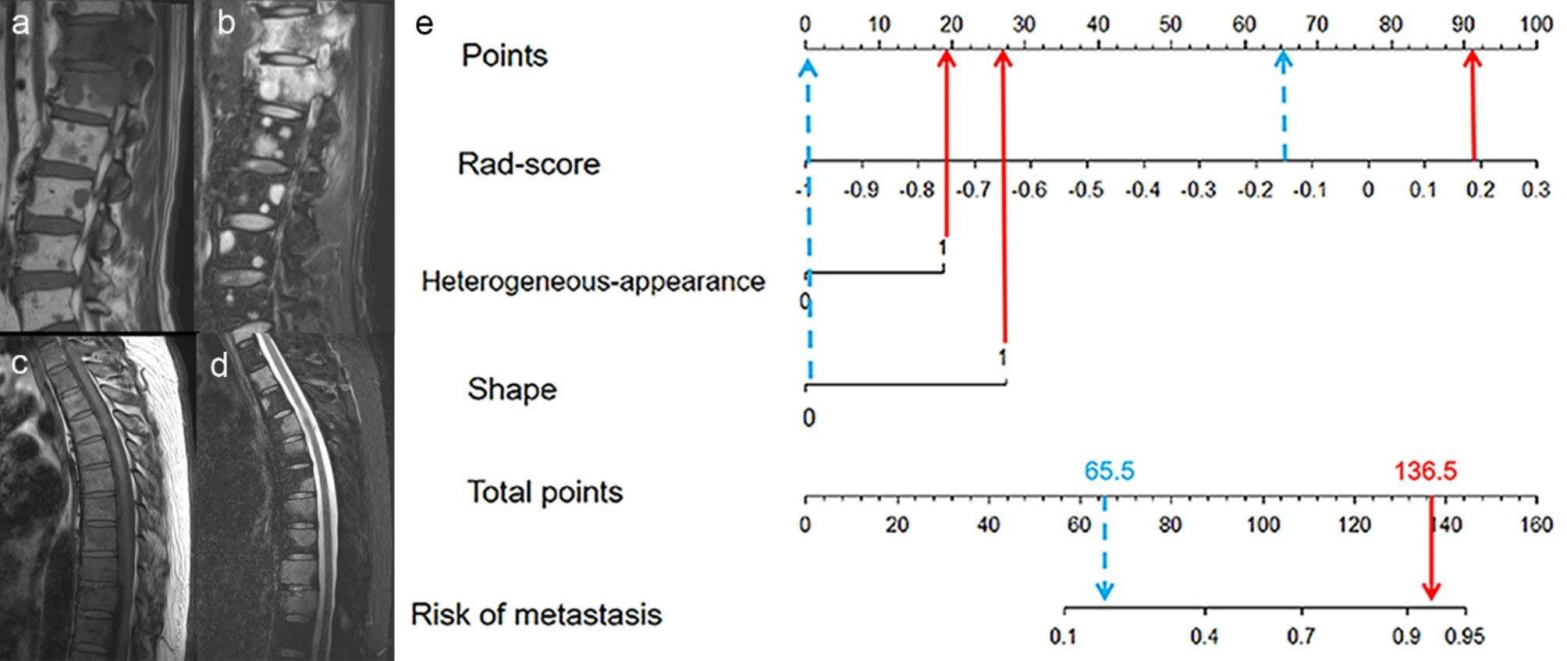
Image examples of typical myeloma and metastasis, and application of nomogram to predict probability of metastasis. Nomogram shows points assigned for each predictor. Total number of points is calculated by adding points assigned for all variables and is then used to determine corresponding risks of metastasis. **(a, b)** MRI shows multiple lesions in the spine. And some of the lesions presents heterogeneous appearance and irregular shape on MRI. Patient was diagnosed with metastasis by pathological evaluation. The experienced radiologist correctly rendered diagnosis of metastasis. **(c, d)** MRI shows multiple lesions in the spine. And some of the lesions presents homogenous appearance and regular shape on MRI. Patient was diagnosed with myeloma by the International Myeloma Working Group Diagnostic Criteria. The experienced radiologist correctly rendered diagnosis of myeloma. **(e)** Nomogram shows determination of risk of metastasis in both patients who had multiple lesions on MRI. For patient in **a, b** (red arrows), nomogram yields total of 136.5 points and corresponding risk of metastasis of greater than 0.9. For patient in **c, d** (blue arrows), nomogram yields total of 65.5 points and corresponding risk of metastasis of less than 0.4. Thus, nomogram rendered correct diagnosis in both patients

**Fig. 6:**
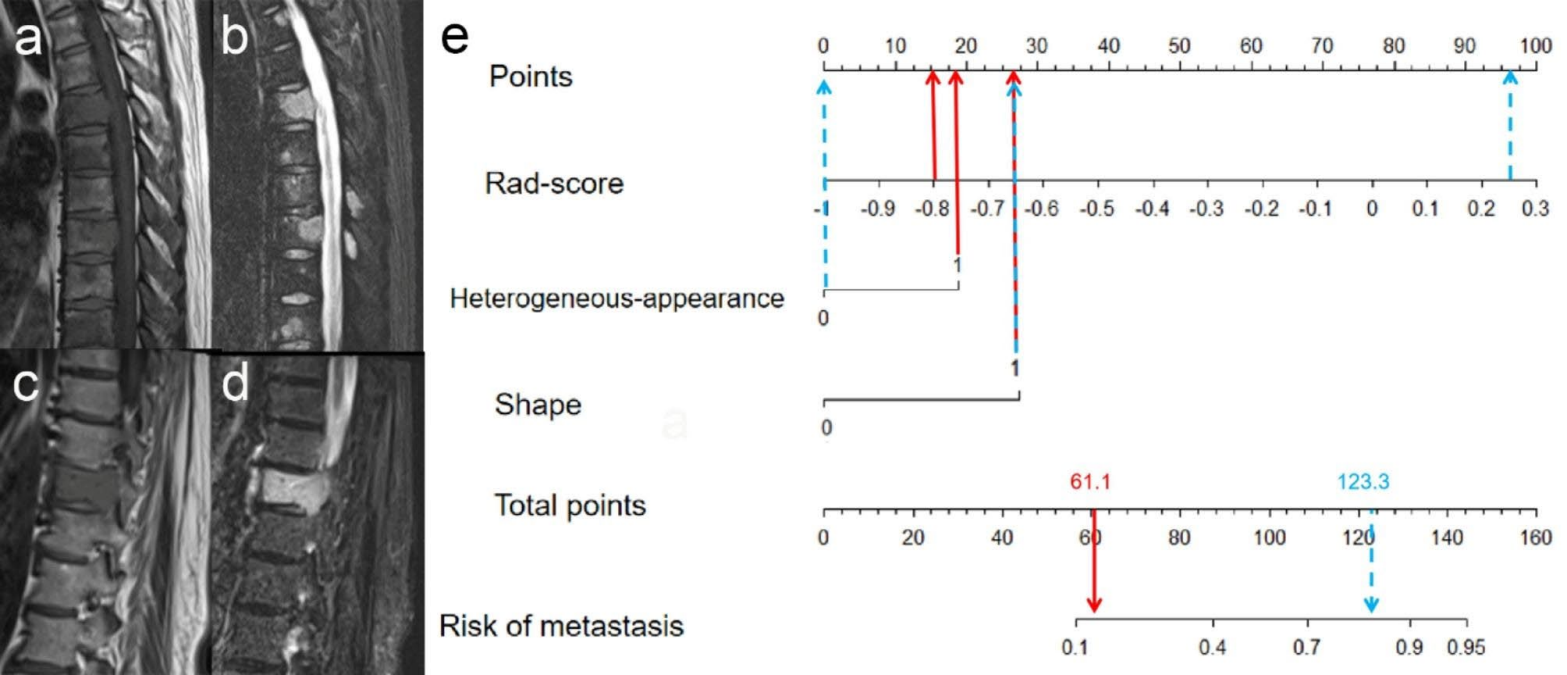
Image examples of myeloma and metastasis which were misdiagnosed by radiologist, and application of nomogram to predict probability of metastasis. **(a, b)** MRI shows multiple lesions in the spine. And some of the lesions presents heterogeneous appearance and irregular shape on MRI. Patient was diagnosed with myeloma by the International Myeloma Working Group Diagnostic Criteria. The experienced radiologist incorrectly rendered diagnosis of metastasis. **(c, d)** MRI shows the single lesion in the spine, which presents homogenous appearance and irregular shape on MRI. Patient was diagnosed with metastasis by pathological evaluation. The experienced radiologist incorrectly rendered diagnosis of myeloma. **(e)** Nomogram shows determination of risk of metastasis in both patients. For patient in **a, b** (red arrows), nomogram yields total of 61.1 points and corresponding risk of metastasis of less than 0.4. For patient in **c, d** (blue arrows), nomogram yields total of 123.3 points and corresponding risk of metastasis of greater than 0.7. Thus, nomogram rendered correct diagnosis in both patients

The AUC values of the radiomics nomogram and radiomics signature model were higher than that of radiologist in training set (both *p* < 0.05). The AUC values of the radiomics nomogram model were higher than that of radiologist in validation and external test sets (both *p* < 0.05). However, there were no differences between the AUC values of the radiomics signature model and those of radiologist in validation and external test sets (both *p* > 0.05). Figures 5 and 6 show MRI images in representative patients with myeloma and metastasis, along with the interpretations by the two radiologists and nomogram results in these patients.

## Discussion

The accurate differentiation of metastasis and multiple myeloma is of great clinical significance, because their treatment decision and prognosis differ according to lesion nature. Spinal metastasis and multiple myeloma share many overlapping imaging characteristic, especially multiple osteolytic features, making the differential diagnosis rather difficult by conventional imaging modalities. In this study, we developed the radiomics nomogram for distinguishing metastasis from multiple myeloma with an AUC of 0.856, 0.853 and 0.762, respectively, in the training set, validation set, and external test set.

Sufficient clinical and imaging information facilitate the correct distinction between spinal metastasis and multiple myeloma. The obvious primary tumor or tumor history contributed to the diagnosis of spinal metastasis [14]. Additionally, quantifying M-protein in the serum and urine are valuable for the diagnosis of multiple myeloma [15]. In this study, compared with multiple myeloma, spinal metastasis had a higher prevalence of irregular shape (84.7% versus 56.9%; *p* < 0.001), which could be because spinal metastasis in this study tends to show higher infiltration into the surrounding tissue than multiple myeloma. And spinal metastasis showed a more heterogeneous signal appearance (75.0% versus 62.9%; *p* = 0.024) than multiple myeloma, which is consistent with previous studies [16]. We speculate that these results may be related to histologic features of multiple myeloma, which has a compact arrangement of myeloma cells and small interstitial space, leading to lower heterogeneity [17]. In the current study, multiple logistic regression analysis revealed that heterogeneous appearance and shape were independent predictors. However, the clinical factor model did not achieve high AUC (0.715 in the training set; 0.692 in the validation set; 0.540 in the external test set) for differentiating spinal metastasis and multiple myeloma.

Previous studies have investigated the relationship between the spinal metastasis and multiple myeloma using imaging tools [18, 19]. Li et al. [18] retrospectively analyzed 344 patients with multiple myeloma and bone metastasis by position emission tomography and showed the SUV_max_ values of multiple myeloma (1.6 ± 0.7) were lower than that of spinal metastasis (5.5 ± 2.7; *p* = 0.000). The best cutoff value of SUVmax for differentiating multiple myeloma and spinal metastasis was 2.65 (sensitivity 86.1% and specificity 94.7%; *p* = 0.000) [18]. However, due to its high costs, position emission tomography is not always available in clinical settings, which limits its wide application. Moreover, Lang et al. [19] applied dynamic contrast-enhanced MRI to differentiate between spinal metastasis and multiple myeloma, showing that the wash-out pattern was significantly higher in the myeloma group than the metastatic group (9/9 = 100% vs. 12/22 = 55%, *p* = 0.03). However, Lang’s study was based on small sample size and increasing scanning time. Previous reports attempted to distinguish spinal metastasis from multiple myeloma using diffusion weighted magnetic resonance imaging [5, 6]. Xing et al. [5] studied 53 metastasis and 16 myeloma patients who underwent MRI with 10 b-values and found that the ADC, D, and α values of metastases were higher than those of myeloma, whereas the D* value was lower than that of myeloma (*p* < 0.05). However, the region of interest of this study was placed on the solid component of the tumor to calculate the average value, which may not be appropriate for assessing tumor heterogeneity.

Over recent years, radiomics analyses have been increasingly applied to the tumor diagnosis, grading, and prognostic evaluation [8–10]. Radiomics features may be associated with pathology and genome [20]. Successful applications of radiomics in spine have been reported in differentiating benign spinal tumors from malignant tumors, and discriminating primary spinal tumors and metastases [11, 21]. Vannier et al. [21] developed a radiomics model to differentiate bone islands from spinal osteoblastic bone metastases in CT, resulting in an AUC of 0.96. Previous investigations have shown that radiomics analysis can differentiate between spinal metastasis from multiple myeloma [12, 22]. Yildirim et al. [22] applied the CT histogram analysis to differentiate the multiple myeloma from lytic bone metastases. In histogram analysis, minimum, median, and maximum gray level parameters were found to be significantly higher in lytic bone metastases (*p* < 0.001) [22]. However, the modality of examination in their study was CT. CT scan has radiation and is less visible than MRI for soft tissue [23]. In our study, we chose MRI modality. MRI is considered an ideal initial screening modality for patients with suspected spinal multiple myeloma and metastasis due to its excellent tissue contrast [24, 25]. In addition, bone marrow infiltration can be visualized by MRI even before lytic changes occur [26]. A previous study showed that the MRI radiomics model could well differentiate metastasis from multiple myeloma [12]. However, it mainly focuses on influence of features number and the limited sample were collected from one center. In our study, we analyzed 312 patients from multiple centers and developed different models to differentiate spinal metastasis from multiple myeloma, providing more comprehensive diagnosis analysis.

In our study, we firstly developed the radiomics nomogram for distinguishing metastasis and multiple myeloma. The nomogram analysis incorporating radiomics signatures and clinical factors can provide more synthetic evidence than conventional approaches, with an AUC of 0.856, 0.853 and 0.762, respectively, in the training set, validation set, and external test set. In addition, compared with most previous studies using data from only one center, patients in external test set in our study were from different hospitals, yielding more reliable results. Moreover, we showed no significant difference in AUC values of radiomics nomogram model between the validation set and external test set. Our radiomics analysis was based on conventional non-contrast MRI, including T1WI and FS-T2WI, which can not add additional scanning time and can be suitable for patients with renal dysfunction or adverse reactions to contrast injection. However, it is possible that differentiation ability could be improved by including advanced MRI image information, such as contrast images and diffusion weight images, which should be further investigated.

Previous studies reported that different MRI scanners with different MRI protocols can lead to inconsistencies in quantitative measurements from these images, which could influence features dimension and reproducibility [27]. And several a successive study has demonstrated that the feature reproducibility can be improved when a image normalization to muscle tissue is applied [28]. Thus, in our study, in order to eliminate the influence of dimension between features and make the intensity information consistent, the images were normalized before analysis, which could be helpful to eliminate the interference caused by the inconsistent image quality caused by different MRI manufactures or different MRI protocols. In addition, the radiomics signature model show a very similar, even slightly better performance in the external multicentric test set (AUC 0.754) compared to the internal validation set (0.743). This may be because the data sample distribution is more balanced (metastasis vs. myeloma: 52 patients vs. 49 patients) in external test set with stronger generalization compared to the internal validation set (metastasis vs. myeloma: 44 patients vs. 21 patients).

Some limitations of this study should be noted. First, it is a retrospective study, so further prospective research is required. We are now following up these patients to assess their prognosis. And the present retrospective study is fundamental to prospective research. Second, MRI acquisition parameters were not consistent due to multi-institutional nature of the study. Third, the lesions were multiple, however, our ROI acquisition was only for the largest lesion. The pathology was record from the largest spinal lesion, and it was considered to reflect the lesion characteristics more accurately, so the largest lesion was selected for radiomics analysis. Fourth, our study included only radiomics analysis of focal lesions but not surrounding tissue. A prior study demonstrated that in an MRI bone marrow radiomics analysis, focal myeloma pattern and diffuse myeloma pattern lead to distinctive radiomics signatures, and several radiomics features from the surrounding tissue was valuable for assessing the myeloma [29]. Further researches about surrounding tissue of lesions with a larger sample size and more detailed clinical and radiomics data are warranted.

## Conclusion

In conclusion, our study developed a MRI-based radiomics nomogram that can predict whether a bone lesion is rather a myeloma lesions or rather a bone metastasis with a moderate to good performance. The diagnostic performance of the radiomics nomogram was superior to radiomics signatures model and clinical factors model. Therefore, it was presented as a non-invasive and valuable method for differentiating between spinal metastasis and multiple myeloma.

## Electronic supplementary material

Below is the link to the electronic supplementary material.

Supplementary Material 1

## Data Availability

The data used and analyzed during the current study are available from the corresponding author on reasonable request.

## Abbreviations

ANOVA: Analysis of variance
AUC: Area under the curve
DCA: Decision curve analysis
FS-T2WI: Fat suppressed T2-weighted imaging
ICC: Intraclass correlation coefficient
LASSO: Least absolute shrinkage and selection operator
ROC: Receiver operator characteristic
ROI: Regions of interests
T1WI: T1-weighted imaging
TE: Echo time
TR: Repetition time
