# Supplementary material for: An MRI-based radiomics nomogram for differentiating spinal metastases from multiple myeloma
Source: Cancer Imaging. 2023 Jul 24;23:72. doi: 10.1186/s40644-023-00585-4 (PMC10367256; doi:10.1186/s40644-023-00585-4)
Supplement: Supplementary file 1 — Supplementary Material 1 [file 40644_2023_585_MOESM1_ESM.doc]

**Supplementary Table 1** Optimal features of radiomics model.

| Variables | Sequence | Types | Radiomics features | Coefficient |
| --- | --- | --- | --- | --- |
| A | FS-T2WI | GLRLM | wavelet-LHH_glrlm_LongRunLowGrayLevelEmphasis | 0.064 |
| B | T1WI | GLRLM | exponential_glrlm_RunLengthNonUniformityNormalized | -0.016 |
| C | T1WI | GLRLM | gradient_glrlm_RunLengthNonUniformityNormalized | -1.83e-17 |
| D | T1WI | GLRLM | square_glrlm_RunLengthNonUniformityNormalized | -1.22e-17 |
| E | T1WI | GLRLM | lbp-2D_glrlm_RunLengthNonUniformityNormalized | -1.22e-17 |
| F | T1WI | SHAPE | original_shape_Maximum2DDiameterColumn | 0.002 |
| G | FS-T2WI | GLDM | wavelet-LHL_gldm_SmallDependenceEmphasis | -0.013 |
| H | FS-T2WI | GLCM | wavelet-HHH_glcm_Imc1 | 0.011 |
| I | FS-T2WI | GLCM | wavelet-HHH_glcm_DifferenceVariance | 0.023 |
| J | T1WI | First-order | wavelet-HHH_firstorder_90Percentile | -0.029 |
| K | FS-T2WI | GLRLM | wavelet-HLL_ngtdm_Busyness | 0.014 |
| L | T1WI | GLRLM | exponential_glrlm_RunVariance | 0.015 |
| M | FS-T2WI | First-order | wavelet-LHL_firstorder_Skewness | -0.020 |
| N | T1WI | First-order | wavelet-LHL_firstorder_Minimum | 0.007 |
| O | FS-T2WI | GLRLM | wavelet-LLH_glrlm_ShortRunEmphasis | -0.003 |
| P | T1WI | First-order | wavelet-LHL_firstorder_Median | -0.001 |
| Q | FS-T2WI | First-order | wavelet-HLL_firstorder_90Percentile | 0.032 |
| R | FS-T2WI | GLDM | wavelet-LHH_firstorder_Kurtosis | 0.006 |
| S | FS-T2WI | GLDM | original_gldm_HighGrayLevelEmphasis | 0.026 |
| T | FS-T2WI | GLDM | logarithm_gldm_HighGrayLevelEmphasis | 1.22e-17 |
| U | FS-T2WI | GLDM | squareroot_gldm_HighGrayLevelEmphasis | 1.22e-17 |
